# Evaluating the Role of Social Media in Veterinary Anatomy and Clinical Education: A Student-Based Study

**DOI:** 10.3390/vetsci12111098

**Published:** 2025-11-18

**Authors:** Ebru Eravci Yalin, Simge Özüner, Zeynep Nilüfer Akçasız, Sevim Güllü, Ozan Gündemir

**Affiliations:** 1 Department of Surgery, Faculty of Veterinary Medicine, Istanbul University-Cerrahpasa, 34320 Istanbul, Türkiye; ebrueravci@gmail.com; 2 Institute of Graduate Studies, Istanbul University-Cerrahpasa, 34320 Istanbul, Türkiye; ozunersimge@gmail.com (S.Ö.); zeynep.akcasiz@iuc.edu.tr (Z.N.A.); 3 Faculty of Sport Sciences, Istanbul University-Cerrahpasa, 34320 Istanbul, Türkiye; 4 Department of Anatomy, Faculty of Veterinary Medicine, Istanbul University-Cerrahpasa, 34320 Istanbul, Türkiye

**Keywords:** veterinary education, social media, student perceptions, anatomy teaching, clinical training, digital professionalism, educational technology, veterinary curriculum

## Abstract

Social media is increasingly present in higher education, particularly in fields that rely on visual learning such as veterinary anatomy and clinical sciences. This study investigated how veterinary students perceived the use of social media for educational and professional purposes, with specific attention to its role in learning anatomy and observing clinical procedures. An online cross-sectional survey was administered to students across different academic levels at a veterinary faculty. The questionnaire included demographic questions and eight Likert-scale items addressing the educational value of social media, its role in accessing learning materials, engagement with professional visuals, and ethical concerns regarding the sharing of clinical or surgical content. Data were analyzed using one-way ANOVA and post hoc Tukey tests to assess differences by academic year, age group, and daily social media usage. Analysis showed that students in earlier academic years generally held more favorable views on the use of social media for accessing learning materials and understanding complex subjects. Students in advanced years expressed greater ethical concern, particularly about sharing surgical or clinical videos online. Daily social media usage showed limited influence on general perceptions, though some variation appeared in topic-specific responses. The results suggest that veterinary programs may benefit from structured educational components on digital professionalism and responsible media use to better align student engagement with ethical standards and educational objectives.

## Simple Summary

Veterinary students are increasingly using social media platforms as part of their learning process. These tools provide access to videos, images, and explanations that can help students understand complex subjects like anatomy and surgery. While extensive research has examined students’ perceptions of educational uses of social media in higher education, evidence remains comparatively limited for veterinary education, particularly in anatomy and clinical learning, where visual–procedural content and professional/ethical standards intersect. In this study, students from a veterinary faculty were surveyed to explore their opinions on the benefits and challenges of using social media in their studies. The results showed that students in the earlier years of their education had more positive views, especially regarding the usefulness of social media in accessing learning materials and engaging with visual content. In contrast, those in advanced academic years were more cautious, particularly about the ethical concerns surrounding the sharing of clinical or surgical videos. These findings suggest that veterinary education could benefit from including guidance on the responsible and effective use of social media. Understanding student perspectives can help institutions better support digital learning while promoting ethical and professional behavior online.

## 1. Introduction

Social media platforms have rapidly evolved from tools of personal communication into powerful instruments for information dissemination, collaboration, and learning [1,2,3]. In higher education especially, platforms like Facebook, Twitter, YouTube, and Instagram are increasingly used to enhance student engagement, communication, and peer interaction [4,5,6]. Empirical studies show that social media use can improve student–student and student–teacher interactions, elevate motivation, and foster academic engagement—benefits that are particularly strong when integrated into structured learning activities rather than being used informally [7,8]. Systematic reviews reinforce this view, highlighting social media’s role in promoting collaborative learning, self-directed learning, and improved academic performance, although acknowledging challenges related to distraction and privacy concerns [8,9].

In veterinary education, particularly in anatomy and surgery, social media platforms had emerged as valuable adjuncts to traditional teaching methods [10,11,12]. These disciplines relied heavily on visual-spatial understanding and procedural exposure, which were partially addressed through digital content previously shared online. For instance, during the COVID-19 pandemic, YouTube had been utilized to deliver veterinary anatomy and surgical content asynchronously, so that students could observe dissection and operative videos when hands-on access was restricted [13]. In another initiative, a cohort of first-year veterinary students participated in collaborative canine abdominal dissections where they documented their procedures with video and photographs, compiled them into a shared online wiki, and performed better in anatomy assessments compared to peers who had not engaged with the digital portfolio [14]. Additionally, narrative and systematic reviews had shown that social media posts—such as Instagram visuals and YouTube modules—had supported anatomy learning by enhancing student confidence and peer learning, although educators raised concerns about privacy, ethical standards, and the variable educational quality of publicly available videos. These findings indicated that social media had facilitated access to rare clinical cases and high-quality anatomical demonstrations, thereby extending educational opportunities beyond the dissection room into widely accessible digital spaces [15,16,17].

Social media has emerged as a widely adopted tool in higher education, offering students opportunities for collaborative learning, self-directed study, and greater engagement with peers and instructors [6,18]. In veterinary education, particularly in anatomy and surgery where visual and procedural learning is central, platforms like YouTube and Instagram have allowed students to access dissection videos, surgical demonstrations, and rare clinical cases, complementing hands-on experience, especially during the COVID-19 pandemic [19,20]. While such materials are often perceived as valuable learning aids, concerns remain regarding their accuracy, source reliability, and alignment with professional and ethical standards. Despite the growing literature on digital tools in education, few studies have explored how veterinary students themselves balance the educational benefits of these resources with their ethical implications, particularly as their clinical exposure and professional awareness evolve across academic years, a gap this study seeks to address.

At the same time, the literature underscores learners’ ambivalence: students frequently cite distraction, privacy, and the blurring of personal–academic boundaries as salient concerns, even as they acknowledge the benefits of visual and collaborative content [6,8,9,18]. These issues are amplified in health-professional settings, where ethical and professional considerations are paramount. Guidance for anatomists emphasizes informed consent, confidentiality, appropriate contextualization, and professional decorum when creating or sharing cadaver images and surgical content online [15]. Parallel evidence from medical and health-professional education documents both the pedagogical potential of social media and the importance of maintaining online professionalism [16,17,18]. Moreover, studies of moral sensitivity and ethics education suggest that professional awareness tends to increase with clinical exposure and training—an effect especially relevant when senior students evaluate clinical or surgical materials online [21,22].

There is, in fact, a substantial body of research on students’ perceptions of social media in higher education. Empirical and review studies identify pedagogical, social, and technological affordances—greater access to materials, opportunities for collaboration, and increased engagement—when implementation is purposeful and aligned with learning goals [6,7,8,9,18,23,24]. Complementing these general findings, cross-disciplinary work shows that students view social media as an interactive, informational learning tool [25]; platform-specific interventions (e.g., LinkedIn) can strengthen students’ professional profiles and perceived employability skills [26]; and perceptions of social-media teaching tools in higher education are generally positive albeit context-dependent [27].

Context also matters. In developing-country settings, social media has been leveraged to sustain formal academic communication when institutional platforms were unavailable or unstable, enabling timely announcements, coordination, and learning support across cohorts and staff [28]. Yet debates persist about whether and how to use social media in higher education in such contexts, with studies weighing accessibility and cost-effectiveness against governance, privacy, and pedagogical consistency [29]. During the pandemic, e-learning research from India documented extensive student reliance on social media to access course materials, clarify doubts, and complement synchronous instruction [30]. Complementary comparative work also examined Microsoft Teams versus social network sites, showing that learners’ experiences and satisfaction can differ across modalities and that formal collaboration platforms and open social networks may serve distinct, complementary roles within a single course sequence [31]. Together, these findings reinforce that social media’s educational value is shaped by infrastructure, policy, and course design—factors that vary across institutions and regions.

Within veterinary education—especially in anatomy and surgery/clinical training—these opportunities intersect with ethical and professional obligations. The inherently visual–spatial and procedural character of these disciplines makes them well-suited to video- and image-based learning [10,11,12,19,20], yet increasing clinical exposure and client-facing responsibilities can heighten students’ ethical sensitivity as they progress through the curriculum [21,22]. Although the higher-education literature on social media and student perceptions is extensive [6,7,8,9,18,19,20,21,22,25,26,27,28,29,30,31], comparatively fewer studies have examined how veterinary students themselves evaluate the educational and ethical dimensions of social media specifically within anatomy and clinical contexts, where visual media and professional standards converge most strongly. Addressing this discipline-specific gap can inform both pedagogical design (e.g., how to harness visual content effectively) and professionalism education (e.g., how to uphold ethical norms when engaging with clinical material online) [15,16,17,21,22].

Grounded in this literature, the present study examines veterinary students’ perceptions of social media with a specific focus on anatomy and clinical learning. We test whether perceptions vary by academic year and age group—reflecting differences in clinical exposure and professional awareness—and whether daily social media use relates to topic-specific attitudes (e.g., acceptance of sharing surgical videos). Our goal is to provide discipline-relevant evidence that can guide the responsible and effective integration of social media into veterinary curricula, balancing accessibility and visual engagement with ethical and professional standards [15,18,21,22,28,29,30,31].

## 2. Materials and Methods

### 2.1. Study Design

This study was designed as a cross-sectional survey to evaluate the impact of social media on veterinary education and to assess students’ perceptions of the academic and professional use of social media. The study targeted students at Istanbul University-Cerrahpaşa, Faculty of Veterinary Medicine, encompassing responses from participants across different academic years.

The study targeted the entire population of veterinary students at Istanbul University-Cerrahpaşa (N = 879). The sample consisted of 227 students who participated voluntarily in an online survey. This approach can be described as a convenience sample or voluntary response sample, since participants self-selected into the study. All class years were invited, ensuring a broad cross-section of the population. While this non-probability sampling method is pragmatic in an educational setting, it may introduce some self-selection bias (i.e., students with stronger opinions about social media might be more likely to respond). Nevertheless, the obtained sample (227 respondents) constitutes about 26% of the entire student body, which is a substantial coverage.

Sample Size Adequacy: The adequacy of the 227-student sample was evaluated against standard sample size criteria for a finite population. Using a 95% confidence level and a 5% margin of error as benchmarks, one can calculate the recommended sample size for a population of 879. According to widely used formulas (e.g., Cochran’s formula with finite population correction) or published tables, a sample of approximately 265–270 students would be needed for 95% confidence and ±5% precision in a population of this size. For instance, Krejcie and Morgan’s classic sample size table suggests around 269 respondents for a population of ~900 at 95% confidence and 5% error margin [5]. In our study, 227 volunteers were obtained, which is slightly below this ideal target. This shortfall is relatively minor—the achieved sample still provides a high degree of confidence in the results. Statistically, using 227 instead of 270 increases the margin of error only a bit (to roughly ~5.6% at 95% confidence, by our calculations). In other words, the precision is only marginally affected. Given practical constraints in survey research (where a 100% response rate is rarely feasible), a sample of 227 is reasonably close to the optimum and can be deemed sufficient for analysis. Similar studies in education have accepted comparable sample sizes for populations under 1000, especially when achieving a truly random sample is difficult. We acknowledge that the sampling was voluntary; however, the large fraction of the population captured helps mitigate concerns about representativeness. Overall, with 227 respondents the study meets the criteria of an acceptable sample size at the 95% confidence level (with about ±5–6% margin of error), which supports the reliability of the survey findings. Future studies could aim for random sampling to further strengthen generalizability, but the current sample size is justified statistically and in line with sample size recommendations for the given population [32].

### 2.2. Participants

The study included a total of 227 students from Istanbul University-Cerrahpaşa, Faculty of Veterinary Medicine. Participants represented various academic years: 93 students from the 1st year, 54 from the 2nd year, 21 from the 3rd year, 19 from the 4th year, and 40 from the 5th year. Students from all academic years were included on a voluntary basis, and data collection was conducted via an online survey. Only students officially enrolled at Istanbul University-Cerrahpaşa, Faculty of Veterinary Medicine were eligible to participate, and all participants joined on a voluntary basis. All participants completed an informed consent form prior to starting the survey, confirming that they had been informed about the study’s purpose, voluntary nature, and anonymity of responses.

The study employed voluntary sampling, with participation open to all officially enrolled students across the five academic years. Although recruitment was based on self-selection, the final sample included students from each academic year in proportions roughly aligned with the overall student distribution at the faculty, supporting the representativeness of the data.

### 2.3. Data Collection

Data were collected between November 2024 and the end of February 2025. The survey was prepared using the online platform Google Forms and distributed to students through email and social media groups [33,34]. The questionnaire was designed to gather demographic information and measure students’ perceptions of social media. No personal information was collected during the data collection process, and the anonymity of the participants was ensured.

### 2.4. Survey Structure

The questionnaire was specifically developed for this study, and its items were based on social media use in higher, medical, and veterinary education, and then adapted to the veterinary context. First, an item pool was created to cover three domains that are frequently discussed in the literature, educational value, visual/illustrative support, and ethical–professional considerations, and this pool was reviewed by two faculty members for relevance and clarity. Minor wording changes were made after this expert review. It consisted of two main sections.

The first section collected demographic data, including age (18–21 or 22+), academic year, and daily time spent on social media (categorized as 0–1 h, 2–3 h, or 4+ h).

The second section evaluated students’ perceptions of social media through eight statements rated on a five-point Likert scale (1 = strongly disagree to 5 = strongly agree). The eight perception items were grouped into three thematic categories: (i) Educational Value, (ii) Visual/Engagement with content, and (iii) Ethical/Professional considerations.

Questions 4 (“No harm in sharing surgical videos on social media”) and 5 (“Sharing surgical processes benefits professional development”) particularly highlight the ethical dimension of students’ attitudes toward social media use. These items reflect students’ awareness of professional responsibility and the potential ethical implications of sharing surgical content online.

To assess the reliability and validity of the instrument, several procedures were undertaken. A pilot test with 10 students confirmed the clarity of the items, resulting in only minor wording revisions. Internal consistency reliability was evaluated using Cronbach’s alpha, which demonstrated high reliability for the overall eight-item scale (α = 0.84). Subscale reliability was acceptable for the Educational Value domain (α = 0.72), marginal for Visual Engagement (α = 0.69), and lower for Ethical Awareness (α = 0.63), which is expected given the limited number of items per subscale. These results indicate that the instrument provides a reliable measure of students’ perceptions while capturing distinct dimensions relevant to educational, visual, and ethical aspects of social media use.

Item Q8 was designed to gauge students’ interest in participating in an elective course or seminar, potentially integrated into the veterinary curriculum, that would address the professional and responsible use of social media as an educational and clinical tool.

### 2.5. Statistical Analysis

The collected data were analyzed using the R (version 4.4.2) programming language [35]. The statistical analyses were conducted to test the study’s two primary hypotheses:

(1) That students in earlier academic years would report more positive perceptions of social media as a learning tool in anatomy and clinical education compared to those in later years;
(2) Those younger students (18–21 years) would express fewer ethical concerns regarding clinical or surgical content on social media than older students (22+ years).

To address these hypotheses, descriptive statistics were first used to summarize the distribution of demographic variables, including academic year, age, and daily social media usage time. Differences between groups were then assessed using One-Way Analysis of Variance (ANOVA) [36] for each of the eight Likert-scale items, grouped under the three thematic domains of Educational Value, Visual Engagement, and Ethical Awareness. For any item where ANOVA indicated significant differences, the Tukey HSD post hoc test was employed to identify which specific groups differed [37].

We conducted an exploratory factor analysis (EFA) on the 8 Likert-type items using principal-axis factoring with promax (oblique) rotation. Sampling adequacy and the factorability of the correlation matrix were verified: Kaiser–Meyer–Olkin (KMO) = 0.837 and Bartlett’s test of sphericity, χ^2^(28) = 713.98, *p* < 0.001. Factor retention considered eigenvalues > 1, scree plot, and theoretical interpretability. Items were retained when their primary loading ≥ 0.40 and their largest cross-loading < 0.30. For reliability/validity, we report Composite Reliability (CR) and Average Variance Extracted (AVE) for the item sets that satisfied the retention rule; oblique factor correlations (Φ) were also inspected to reflect conceptual relatedness among dimensions.

A significance level of *p* < 0.05 was adopted to determine statistical significance. Mean responses and confidence intervals were calculated for each item by academic year, age group, and social media usage time. To enhance interpretability, results were visualized using the ggplot2 package in R (version 4.4.0), including boxplots and bar charts to highlight group differences in students’ perceptions regarding the educational benefits and ethical considerations of social media use.

## 3. Results

### 3.1. Measurement Model: Exploratory Factor Analysis

An exploratory factor analysis (EFA) was conducted on the eight Likert-scale items to evaluate the dimensional structure of students’ attitudes toward the use of social media in veterinary education. The principal-axis factoring method with promax (oblique) rotation was applied, as correlations among latent dimensions were expected. Sampling adequacy and the factorability of the correlation matrix were satisfactory (KMO = 0.837) and Bartlett’s test of sphericity was significant (χ^2^(28) = 713.98, *p* < 0.001), indicating that the data were suitable for factor analysis.

The analysis revealed a three-factor solution, consistent with the conceptual framework:

F1: Ethical and professional awareness & sharing
F2: Educational value/visual–procedural support
F3: Professional sharing & adoption inclination

After applying a conservative retention rule (primary loading ≥ 0.40, maximum cross-loading < 0.30), two items loaded clearly on F1, while the other factors contained items with moderate cross-loadings or lower loadings that did not meet the strict thresholds. These two items reflected attitudes toward the ethical and professional implications of sharing clinical materials on social media.

Composite reliability and average variance extracted were computed for the retained items. As expected from the limited number of indicators, F1 showed CR = 0.36 and AVE = 0.22, whereas the remaining two factors could not meet reliability criteria under the conservative rule. The oblique factor correlation matrix indicated a moderate positive association between F2 and F3, reflecting that perceived educational value is related to adoption inclination.

### 3.2. Descriptive and Comparative Analysis of Veterinary Students’ Perceptions of Social Media

The class-wise mean scores in the table reveal notable differences in how veterinary students perceive the impact of social media on education and professional development. Overall, Class 1 students exhibited a more positive attitude toward the benefits of social media, consistently providing higher average scores compared to other classes. For instance, in the question “Social media provides useful access to different educational materials,” Class 1 had the highest average score (3.86), while Class 5 scored the lowest (3.30) (Table 1).

Similarly, for the question “Social media makes challenging veterinary education enjoyable through fun content,” Class 1 again provided the highest score (4.20), while Class 5 gave the lowest (3.43). This suggests that as students’ progress through their education, their perception of the positive impact of social media on education becomes less favorable. A similar trend was observed in the question “Professional videos and visuals make it easier for me to understand veterinary topics,” where Class 1 had the highest average (4.30), and Class 5 had the lowest (3.83).

In more professionally focused questions, such as “Sharing treatment and surgical processes on social media benefits my professional development” and “Social media platforms are effective tools for professional knowledge sharing,” Class 5 again provided lower scores compared to other classes. This indicates that students nearing graduation may take a more critical approach to the professional benefits of social media.

Finally, in the question “I am interested in elective courses on ‘social media usage’ taught by experts at faculties for professional purposes,” the distribution of scores across classes was more balanced. However, Class 1 still provided the highest score (3.54). This analysis suggests that perceptions of the role of social media in education and professional development evolve as students’ progress through their studies, with higher years students exhibiting a more critical perspective.

The following findings are based on statistical comparisons between classes, utilizing ANOVA and Tukey HSD post hoc tests to determine significant differences in perceptions of social media in veterinary education (Table 2). In the analysis of the impact of social media on veterinary education, significant differences were observed between classes for certain questions. Notably, Question 4 (“I see no harm in sharing surgical videos on social media”) and Question 5 (“Sharing treatment and surgical processes on social media benefits my professional development”) showed greater variability between classes compared to other questions. For these questions, Class 5 consistently provided lower scores compared to other classes. For instance, when comparing Class 5 to Class 1, the difference in scores for Question 4 was −0.931 (*p* < 0.001), and for Question 5, it was −0.698 (*p* < 0.001), both of which were statistically significant.

Additionally, for Question 1 (“Social media provides useful access to different educational materials”), a significant difference was found between Class 2 and Class 1 (*p* = 0.022). This result suggests that students in Class 2 perceive the benefits of social media for accessing educational materials less positively compared to Class 1.

**Figure 1:**
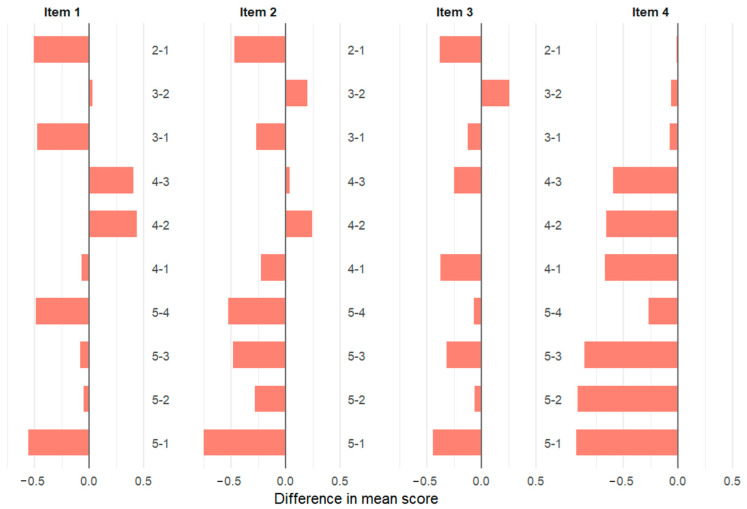
Pairwise comparison of social media items by class (Items 1–4).

**Figure 2:**
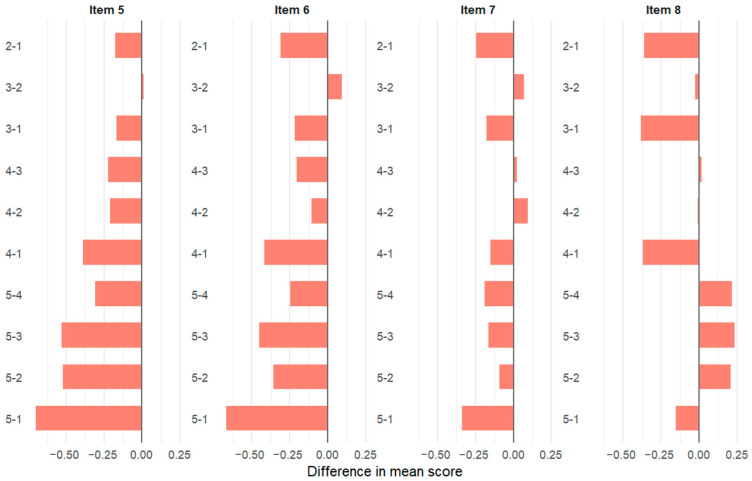
Pairwise comparison of social media items by class (Items 5–8).

Figure 1 and Figure 2 illustrate the pairwise class comparisons obtained from the Tukey HSD test. Each horizontal bar represents the difference in mean scores between two student classes (e.g., 5 − 1 = Class 5 minus Class 1). The direction of the bar (positive or negative) indicates whether the higher-numbered class rated the item more positively or negatively. The length of the bar reflects the magnitude of this difference, with longer bars representing greater differences in mean values. Colored panels correspond to the eight perception questions included in the survey.

Overall, students in Class 5 appeared to have a more critical perspective on the professional benefits of social media. While some differences were also observed between other classes, these were generally smaller or closer to the significance threshold (Figure 1 and Figure 2). The results indicate that perceptions of social media’s professional use and benefits may change as students progress through their education. Differences in responses were particularly pronounced for questions related to the sharing of surgical videos and their professional contributions.

Significant differences were found between age groups regarding perceptions of the impact of social media on veterinary education. Students in the 18–21 age group tended to view social media more positively, whereas those in the 22+ age group demonstrated a more critical perspective. These differences were particularly evident in questions about the entertaining and educational aspects of social media as well as its professional benefits. For example, in the question “Social media makes challenging veterinary education enjoyable through fun content,” the 18–21 age group provided significantly higher average scores compared to the 22+ age group (*p* = 0.002). Similarly, for the question “Professional videos and visuals make it easier to understand veterinary topics,” younger students appreciated the contributions of social media more than their older counterparts, who provided significantly lower scores (*p* = 0.020).

One of the most striking differences appeared in the question “I see no harm in sharing surgical videos on social media.” In this case, the 22+ age group gave significantly lower scores than the 18–21 age group, reflecting a more critical attitude toward sharing surgical videos (*p* < 0.001). A similar pattern was observed for the question “Sharing treatment and surgical processes on social media benefits my professional development,” where the older age group rated the benefits of such content significantly lower than the younger group (*p* < 0.001).

In conclusion, younger students generally perceive the contributions of social media to education more positively, while older students adopt a more critical perspective, especially on sensitive topics such as professional benefits and the sharing of surgical videos. These findings suggest that perceptions of social media’s impact on education and professional development may evolve with age.

According to the analysis results, social media usage time did not have a statistically significant impact on students’ overall perceptions of social media. However, for Question 4 (“I see no harm in sharing surgical videos on social media”), a statistically significant difference was observed among students based on their social media usage time (*p* = 0.0212, ANOVA). Students who used social media for 4 h or more gave the highest scores regarding the sharing of surgical videos. In contrast, students who used social media for 0–1 h gave lower scores, reflecting a more critical perspective on this topic.

Nevertheless, the results of the Tukey HSD post hoc test indicate that no significant differences could be fully determined between specific social media usage groups. For instance, a difference of −0.65 points was observed between students using social media for 0–1 h and those using it for 2–3 h, but this difference was not statistically significant (*p* adj = 0.109). Similarly, the difference between the 0–1 h group and the 4 h or more group was also not statistically significant (*p* adj = 0.630). However, the difference between the 2–3 h group and the 4 h or more group (0.35 points) was close to the threshold for statistical significance (*p* adj = 0.056).

These findings suggest that while social media usage time does not have a clear impact on students’ overall perceptions of social media, it may influence perceptions on specific topics, such as the sharing of surgical videos.

## 4. Discussion

The findings of this study revealed that lower-year veterinary students exhibited more favorable perceptions of social media in both educational and professional contexts compared to students in higher years. First-year students consistently assigned higher scores to items related to the usefulness of social media in accessing educational materials, enhancing engagement with difficult content, and benefiting from professional videos. In contrast, fifth-year students demonstrated a more critical stance, particularly regarding the sharing of surgical and treatment processes. This pattern may reflect the shift in students’ priorities and awareness as they approach professional practice. With increased clinical exposure and ethical sensitivity, higher years students may become more cautious about the educational and professional boundaries of social media use. Additionally, early-year students may rely more heavily on digital content due to limited hands-on experience and a desire to supplement traditional instruction with visual aids and peer-shared resources.

Age-based comparisons showed a similar trend, with younger students (18–21 years) holding more positive attitudes toward social media’s educational role, particularly regarding engaging content and professional visuals. Older students (22+) tended to rate items related to ethical considerations and surgical content sharing more conservatively. This suggests that maturity and exposure to clinical settings may influence perceptions of professional appropriateness in digital spaces. Interestingly, while overall social media usage time did not significantly impact students’ general attitudes, those who used social media for four or more hours per day expressed greater acceptance of sharing surgical videos. This pattern may reflect a higher level of digital immersion and familiarity, potentially leading to a desensitization effect, where repeated exposure normalizes the viewing and distribution of such material. While this could indicate growing comfort with professional content, it also raises questions about whether frequent exposure diminishes sensitivity to the ethical implications of sharing surgical footage. However, the absence of statistically significant differences between specific usage groups limits the strength of this interpretation, and further research is needed to clarify the relationship between social media habits and students’ ethical perceptions.

The students’ critical perspectives were particularly evident regarding the sharing of surgical videos and anatomical content through social media. This attitude appeared to be closely related to ethical concerns. In a study involving veterinary students, participants emphasized that social media posts could lead to unintended consequences concerning professional responsibility and confidentiality [17]. Furthermore, systematic reviews and guideline articles directed at anatomy educators have highlighted that the dissemination of cadaver images and surgical content should always consider key principles such as informed consent, ethical standards, and professional decorum [15]. In this context, the more critical responses observed among higher years students in our study may be interpreted as consistent with increased awareness of ethical boundaries and align well with existing literature.

Ethical concerns surrounding the use of social media in veterinary education are not limited to issues of professionalism; they also reflect potential conflicts between students’ social media habits and their evolving professional identity [38,39,40]. For instance, a study investigating students’ social media behavior noted that posts made online could negatively affect both individual and professional reputations, with many students posting content under a false sense of anonymity [38]. The fact that higher years students in our sample responded more cautiously to ethically sensitive items suggests a growing internalization of professional values.

The findings of this study have important implications for veterinary education and digital literacy training. The results suggest that integrating social media into formal learning environments could enhance students’ engagement and promote collaborative learning when guided by clear ethical and professional standards. Veterinary educators could use social media as a complementary platform for sharing educational materials, facilitating discussions, and building a sense of professional community among students. However, given the ethical concerns expressed by senior students, universities should also incorporate media literacy and professionalism modules into their curricula to ensure responsible and evidence-based use of social media. These findings may guide curriculum developers and policymakers in designing modern veterinary education strategies that balance innovation with ethical responsibility.

This study has several limitations that should be considered when interpreting the findings. First, the data were collected from a single veterinary faculty, which may limit the generalizability of the results to other institutions or regions. Second, the study relied on self-reported survey responses, which may be subject to recall bias or social desirability bias. Third, the use of voluntary sampling may have introduced selection bias, as students with stronger opinions about social media could have been more likely to participate. Fourth, the questionnaire did not ask students to specify which social media platforms they used most frequently, as the study focused on their overall perceptions of social media’s educational and ethical roles rather than platform-specific patterns. While national statistics suggest that Instagram and YouTube are among the most popular platforms for Turkish university students, this contextual information was not directly measured in our dataset. Finally, dedicated e-learning platforms (e.g., Moodle, Google Classroom) were not included in the analysis because they serve distinct educational purposes, even though a few respondents mentioned them in open-ended comments. Future research incorporating platform-specific data and comparisons with e-learning systems would provide a more nuanced understanding of students’ digital learning habits.

In addition, although the study provides valuable insights into veterinary students’ perceptions of social media in education and professional development, its findings should be interpreted with caution due to the relatively low response rate. The limited participation may reduce the generalizability of the results to the entire population of veterinary students. Future research involving a larger and more diverse sample across multiple institutions would help to validate and extend these findings, offering a broader and more representative understanding of how social media influences educational and professional development among veterinary students.

These findings highlight the need for veterinary curricula to deliberately address the educational and ethical dimensions of social media use [10,38,40,41,42]. While younger and early-year students tended to welcome social media as a learning tool, their enthusiasm often lacked the critical awareness of professional and ethical boundaries noted in prior research [40,43]. Conversely, higher-year students demonstrated a more nuanced understanding, likely influenced by their greater clinical exposure and institutional expectations. Integrating vertically structured modules on digital literacy and professionalism, as previously advocated in medical and dental education [41,42], may help veterinary students balance the benefits of social media with responsible and ethical engagement.

## 5. Conclusions

This study revealed that veterinary students’ attitudes toward social media as an educational and professional tool differed significantly by academic year and age. Early-year and younger students tended to view social media more positively for accessing learning materials, professional visuals, and supplementary resources, while senior students demonstrated greater caution, particularly concerning the ethical implications of sharing surgical and clinical content. These findings indicate that digital literacy and professional awareness evolve as students’ progress through their training.

Given these results, integrating structured curricular modules on the responsible and professional use of social media may enhance students’ ability to leverage digital resources while maintaining ethical standards. Such modules could address both the opportunities social media offers for anatomy and clinical education and the potential risks, such as privacy concerns and unverified content. Implementing these strategies may foster more consistent and professional engagement with online learning resources across all academic levels.

Future research should include multi-institutional and longitudinal studies to determine how students’ attitudes and behaviors toward social media evolve over time and to assess the effectiveness of targeted curricular interventions in shaping responsible digital practices.

## Figures and Tables

**Table 1:** Class-Wise Mean Scores of Veterinary Students on Social Media Perception Questions.

Question	Mean	Class 1	Class 2	Class 3	Class 4	Class 5
Social media provides useful access to different educational materials.	3.59	3.86	3.35	3.35	3.79	3.30
Social media makes challenging veterinary education enjoyable.	3.89	4.20	3.70	3.90	3.95	3.43
Professional videos make it easier to understand veterinary topics.	4.06	4.30	3.89	4.20	3.89	3.83
No harm in sharing surgical videos on social media.	4.23	4.45	4.44	4.35	3.79	3.53
Sharing surgical processes benefits professional development.	4.34	4.55	4.37	4.40	4.16	3.85
Social media is effective for professional knowledge sharing.	4.12	4.36	4.06	4.15	3.95	3.70
I learn new information via social media.	4.26	4.41	4.17	4.25	4.26	4.08
Elective courses on social media interest me.	3.35	3.54	3.17	3.15	3.16	3.38

Source: Authors’ own data, collected between November 2024 and February 2025.

**Table 2:** ANOVA Results: Class-Wise Differences in Social Media Perception Among Veterinary Students.

Question	F Value	*p*-Value
1. Social media provides useful access to different educational materials.	6.35	*p* < 0.05
2. Social media makes challenging veterinary education enjoyable.	11.90	*p* < 0.001
3. Professional videos make it easier to understand veterinary topics.	6.07	*p* < 0.05
4. No harm in sharing surgical videos on social media.	24.25	*p* < 0.001
5. Sharing surgical processes benefits professional development.	18.28	*p* < 0.001
6. Social media is effective for professional knowledge sharing.	12.71	*p* < 0.001
7. I learn new information via social media.	3.66	*p* ≈ 0.05
8. Elective courses on social media interest me.	0.608	Not Significant

Source: Authors’ own data, collected between November 2024 and February 2025.

## Data Availability

The raw data supporting the conclusions of this article will be made available by the authors on request.
